# Aromatherapy in Stroke Patients: Is it Time to Begin?

**DOI:** 10.3389/fnbeh.2021.749353

**Published:** 2021-12-08

**Authors:** Marianna Contrada, Antonio Cerasa, Paolo Tonin, Giacinto Bagetta, Damiana Scuteri

**Affiliations:** ^1^S.Anna Institute and Research in Advanced Neurorehabilitation (RAN), Crotone, Italy; ^2^IRIB, National Research Council, Messina, Italy; ^3^Pharmacotechnology Documentation and Transfer Unit, Preclinical and Translational Pharmacology, Department of Pharmacy, Health and Nutritional Sciences, University of Calabria, Rende, Italy

**Keywords:** aromatherapy, pain, autonomic response, neuroprotection, stroke

## Abstract

Stroke is the second largest cause of death worldwide, causing disease with long-term consequences and considerable healthcare costs. The application of new nursing interventions aimed at reducing distressing behaviors and at increasing patient comfort is an important part of the care and, until now, there are no defined guidelines. Aromatherapy has been demonstrated to be efficient in several other neurological disorders for the treatment of somatic and emotional diseases and to promote patient health. In the management of stroke patients, aromatherapy is still in its infancy. The first evidence coming from animal models demonstrated a consistent and reliable neuroprotective effect in reducing cerebral ischemia–reperfusion injury. In the last few years, some preliminary data being to be collected in humans revealed significant influence in reducing patients’ pain and emotional distress. In this perspective study, we sought to summarize, for the first time, the main findings emerging from this new field of study, discussing the future opportunities to be translated into primary care practice.

## Caring for Stroke Survivors: The Complementary Medicine

Stroke is the second leading cause of mortality in the world and one of the most chronic diseases causing great burden in terms of physical, cognitive, and emotional disability (World Health Organization, 2020). Because of the chronic nature of this condition, patients were normally treated with both pharmacological and rehabilitation therapies, but these can be strongly influenced by mood status. Indeed, at a behavioral level, pain and anxiety are the most common complications preventing functional recovery as they can interfere with self-care, balance, ambulation, and quality of life (Snels et al., 2002; Rafsten et al., 2018). In 16%–80% of patients who are hemiplegic after stroke experience shoulder pain at least once during their rehabilitation (Snels et al., 2002), whereas the prevalence of anxiety disorders was detected in 29.3% of patients during the first-year post-stroke (Rafsten et al., 2018).

Currently, licensed medications have limited efficacy against somatic disorders and distressing behaviors (Wainapel et al., 1998). This creates an enormous challenge for professional care systems with the need of defining new treatments for improving the patients’ and caregivers’ quality of life providing substantial relief for emotional disorders. In the last few years, some empirical data have demonstrated that these symptoms could be improved by using complementary and alternative medicine, where the employment of essential oils (aromatherapy) has gained great popularity (Wainapel et al., 1998; Farrar and Farrar, 2020).

Aromatherapy involves the therapeutic use of essential oils as complementary medicine to treat many physical and emotional diseases and to promote patient health (Reis and Jones, 2017). Individual constituents of essential oils can reach the blood, cross the blood-brain barrier, and enter the CNS following oral administration, dermal application, inhalation, intraperitoneal or subcutaneous injection (Cooke and Ernst, 2000). It has widely been demonstrated that aromatic oils (such as lavender, bergamot, or curcuma) have beneficial effects on alleviating mood and mild symptoms of stress-induced disorders such as depression (Komori et al., 1995), anxiety (Lehrner et al., 2000), as well as migraine (Yuan et al., 2021), chronic pain in multiple sclerosis patients (Esmonde and Long, 2008), and behavioral disturbances in dementia (Smallwood et al., 2001). Moreover, aromatherapy can also improve somatic symptoms, such as blood pressure (Perry and Perry, 2006) and respiratory rate in patients before breast surgery (Beyliklioğlu and Arslan, 2019). In particular, lavender has been demonstrated to have analgesic, sedative, and antispasmodic healing properties in oncological (Ozkaraman et al., 2018) and burn patients (Seyyed-Rasooli et al., 2016). Finally, the beneficial effects of essential oils go beyond behavioral symptoms directly influencing neurochemical processes inside the brain. Indeed, it has been demonstrated that Bergamot and Curcuma oils may also have antioxidant and neuroprotective effects on cerebral ischemia (Dohare et al., 2008).

In the last few years, new studies have been made for demonstrating the impact of aromatherapy in alleviating physical and emotional symptoms in stroke patients, but until now a revision of literature is lacking. Animal studies have provided the first consistent biological evidence on how aromatherapy may impact mechanisms underlying the pathogenesis of ischemic stroke. Otherwise, there are few clinical studies in humans, although preliminary data demonstrated an improvement in somatic disorders and distressing behaviors. For this reason, we sought to perform, for the first time, a synthesis of recent advances in this challenging field of study, where preliminary evidence in animal models seems to encourage future clinical studies aimed at demonstrating the translational potential of this complementary medicine in primary care practice of stroke patients. For this perspective article, we only considered randomized controlled studies where the stroke was the only clinical domain investigated and aromatherapy was the main treatment employed.

## Aromatherapy in Animal Models of Stroke

Until now, eight pre-clinical studies have directly evaluated the effects of essential oils on brain stroke (Table 1). What clearly emerged from these studies was that essential oils significantly reduce infarct area in a cerebral ischemia mouse model by promoting neuroprotective mechanisms. We briefly present the main findings of these studies. Dohare et al. (2008) attempted to clarify the neuroprotection effects of Curcuma oil (C.oil) treatment against experimental middle cerebral artery occlusion (MCAo) induced in Sprague–Dawley male rats. Experimental groups for *in vivo* and *in vitro* neuronal studies were divided in: (a) ischemia/reflow (I/R), pretreated with C.oil; (b) ischemia/reflow (I/R), no treatment; and (c) sham-operated control. C.oil with a dose of 250 mg/kg was used as a test agent. A single dose was intraperitoneally administered 30 min before MCAo. The authors demonstrated that C.oil reduced post-ischemic brain neutrophil infiltration and neurodegeneration. Immunohistochemical and Western immunoblot analyses confirmed that C.oil treatment significantly induced a reduction in the expression of nitric oxide synthase and in numbers of apoptotic cells compared to the untreated ischemic group. The authors claimed that C.oil appears to exert its neuroprotective effects by acting at multiple targets in the neural signaling pathways that are activated in ischemic and neurodegenerative brain diseases. Büyükokuroğlu et al. (2003) sought to investigate the neuroprotective effect of Lavandula angustifolia flower (LAF) in mediating glutamate-induced neurotoxicity in rat pups cerebellar granular cell culture. LAF oil was filtrated and, then, lyophilized in a freezer-dryer at 5 μm Hg pressure and −50°C. Seven experimental groups were tested in five culture media considering 20 microscopic neuronal areas: (1) Control group; (2) Glutamate group; (3) Glutamate + extract of LAF at concentration of 10 μg ml^−1^; (4) Glutamate + extract of LAF at concentration of 100 μg ml^−1^; (5) Glutamate + extract of LAF at concentration of 1 mg ml^−1^; (6) Glutamate + extract of LAF at concentration of 10 mg ml^−1^; and (7) extract of LAF at concentration of 10 mg ml^−1^). The authors found that the extract of LAF exerts neuroprotective activity in glutamate-induced neurotoxicity where the most effective dose was at 1 mg ml^−1^. Wang et al. (2012) observed the neuroprotective effects of lavender oil (obtained from the dried flowers) against ischemic brain injury. Kunming mice were randomly divided into six groups: (1) lavender oil group at doses of 50 mg/kg; (2) lavender oil group at doses of 100 mg/kg; (3) lavender oil group at doses of 200 mg/kg; (4) edaravone group (3 mg/kg, intraperitoneal injection. used as the positive control); (5) vehicle group (maize oil, 10 ml/kg, i.g.); and (6) sham group. Drug or maize oil was administered once at 2 h after the onset of ischemia and once a day for 3 days before ischemia. Lavender oil offered neuroprotection in focal cerebral ischemia/reperfusion. Rabiei et al. (2017) found that lavender oil at doses of 200 mg/kg induced significant neuroprotection against brain ischemia by reducing infarct size, inhibiting lipid peroxidation, protein oxidation, and increasing endogenous antioxidant defense. Vakili et al. (2014) employed aromatherapy with lavender oil in an experimental model of stroke using four different experiments aimed at assessing the effects on: (1) infarct size; (2) brain edema; and (3) the presence of malondialdehyde (MDA; a biomarker of lipid per-oxidation and oxidative stress); and (4) the expression of Bcl-2 (an anti-apoptotic protein) and vascular endothelial growth factor (VEGF). In each experiment, rats were randomly divided into different groups (see Table 1 for more details) and underwent experimental MCAo. Overall, they found that treatment with lavender oil (doses of 200 and 400 mg/kg) significantly improved functional outcomes after cerebral ischemia by diminishing brain edema, infarct size. In particular, lavender oil at a dose of 200 mg/kg, increased the activities of glutathione peroxidase, superoxide dismutase, and total antioxidant capacity while also reducing the content of MDA. No effect was reported on the apoptosis pathway. Taken together, these findings suggest that lavender oil alleviates neurological function in rats and promotes neuroprotective activity against cerebral ischemia. The authors proposed that this mechanism may be related to augmentation in endogenous antioxidant defense as a consequence of inhibiting oxidative stress and increasing vascular endothelial growth factors. Using a different essential oil, Trans-cinnamaldehyde (TCA) extracted from the cinnamon powder, Chen et al. (2016) investigated its neuroprotective effect on an animal experimental model of ischemia/reperfusion (I/R)-induced brain injury. Mice were randomly divided into four groups: (1) TCA treatment group at 10 mg/kg dosage; (2) TCA treatment group at 20 mg/kg dosage; and (3) TCA treatment group at 30 mg/kg dosage; (4) the I/R group (ischemia/reperfusion induction, as a control group). TCA was orally administrated 60 min before ischemia surgery. Amantea et al. (2009) demonstrated that TCA essential oil strongly reduced infarct area in the cerebral ischemic area. Similar to Vakili et al.’s (2014) work, Rabiei and co-authors carried out a study to evaluate the neuroprotective effect of the lavender oil on infarct volume using different doses (100, 200 mg/kg body weight). Lavender was intraperitoneally injected for 20 consecutive days and the amount of serum nitric oxide level was measured before and after treatment. To evaluate the specific effect of aromatherapy, rats were randomly divided into seven groups: (1) group of control—ischemia: distilled water treatment and focal cerebral ischemia; (2) sham group: surgery without obstruction; (3) group under ischemic surgery; (4) group undergoing ischemic surgery; (5) intact-control: group under any surgery; did not sit and received only distilled water; (6) Intact100: any surgery; and (7) Intact200: any surgery. Treatment at a dose of 200 mg induced a significant increase of nitric oxide and a decrease in the infarct volume localized in cortical and subcortical brain regions. As previously hypothesized by Vakili et al. (2014), this neuroprotective effect might be related to augmentation in endogenous antioxidant defense, inhibiting the decrease in cerebral blood flows, which, in turn, leads to infarct volume reduction. Huang et al. (2018) attempted to evaluate how curcumin may mediate the underlying neuroinflammation and neurodegeneration mechanisms induced by cerebral ischemia using a well-established MCAo experimental model. Thirty Sprague Dawley male rats were equally and randomly assigned to five different groups: (1) MCAo group; (2) MCAo + curcumin group; (3) LY294002 (an inhibitor of PI3K) + MCAo + curcumin group; (4) anisomycin + MCAo + curcumin group; and (5) control group. Rats in the second group underwent intraperitoneal injection of 200 mg/kg curcumin 30 min after brain injury. The employment of LY294002 (an inhibitor of PI3K) was for evaluating the autophagy pathway, whereas anisomycin (an activator of p38/MAPK), was for evaluating the inflammation pathway. The authors found that treatment with curcumin significantly improved neurological functions, as determined by the rotarod test, and reduced brain damage by attenuating autophagic activities. Finally, Amantea et al. (2009) evaluated the neuroprotective effects of bergamot essential oil (BEO) against ischemic neuronal injury by using an MCAo model of focal brain ischemia. The authors evaluated the neuropathological and immunoreactivity effects on ischemic brain regions. One hour before MCAo, Wistar rats received intraperitoneal administration of BEO. Five experimental groups were employed: (1) Control group; (2) BEO-treated group at concentration of 0.05 ml/kg; (3) BEO-treated group at concentration of 0.1 ml/kg; (4) BEO-treated group at concentration of 0.5 ml/kg; and (5) BEO-treated group at concentration of 1.0 ml/kg. The authors reported that BEO treatment reduces the volume of brain lesions by increasing excitatory amino acid efflux in the ischemic penumbra and promoting a survival cascade of neuroprotective effects mediated by phosphatidylinositol 3-kinase (PI3-K).

**Table 1:** Main findings of preclinical studies assessing the neuroprotection by essential oils against experimental cerebral ischemia.

References	Phenotype	Treatment	Clinical/Biological measures	Experimental design	Main findings
Dohare et al. (2008)	Mice (Ischemia)	Curcuma oil intraperitoneal injection (single dose 250 mg/kg) administrated 30 min before MCAo	Neuroprotective effects	Mice were randomly divided into three groups: 1) ischemia/reflow (I/R), pretreated with C.oil 2) ischemia/reflow (I/R), no treatment 3) sham-operated control	C.oil reduced post-ischemic brain neutrophil infiltration in the ischemic area. Nitric oxide synthase isoforms and the numbers of apoptotic cells were also significantly decreased.
Büyükokuroğlu et al. (2003)	Mice (Cerebellar neurons Cultures)	Lavandula angustifolia flowers (LAF) oil. A lyophilized preparation of LAF with different doses was applied on cultures of cerebellar granular cells extracted from rats.	Neurotoxicity effects	*In vitro* neurotoxicity experiments were performed in seven groups: 1) Control group; 2) Glutamate group; 3) Glutamate + LAF group (10 μg ml^−1^) 4) Glutamate + LAF group (100 μg ml^−1^) 5) Glutamate + LAF group (1 mg ml^−1^) 6) Glutamate + LAF group (10 mg ml^−1^) 7) LAF group (10 mg ml^−1^)	LAF protected the neurons against glutamate-induced neurotoxicity and neural death. The most effective dose was the extract at 1 mg ml^−1^
Wang et al. (2012)	Mice (Ischemia)	Lavender oil intraperitoneal injection administrated once 2 h after the onset of ischemia and once a day for 3 days before ischemia.	Neurotoxicity and Neuroprotective effects	Mice were randomly divided into six groups: 1) Experimental Group1: Lavender oil-treated group (50 mg/kg) 2) Experimental Group2: Lavender oil-treated (100 mg/kg) 3) Experimental Group 3: Lavender oil-treated (200 mg/kg) 4) Experimental Group 4: Edaravone-treated group (3 mg/kg) 5) vehicle group: maize oil, 10 mL/kg 6) Sham group	Lavender oil at doses of 200 mg/kg induced significant neuroprotection against brain ischemia by reducing infarct size, by inhibiting lipid peroxidation, protein oxidation, and with augmentation in endogenous antioxidant defense.
Vakili et al. (2014)	Rats (Ischemia)	Lavender oil intraperitoneal injection administrated at the onset of ischemia.	Neuroprotective effects	**Experiment 1**:To evaluate the effect of Lavender oil on infarct size, 28 animals were equally and randomly divided into four groups: Control group: (*n* = 7) vehicle (MCAo + maize oil, 1 ml/kg) Experimental Group1: (*n* = 7) Lavender oil at 50 mg/kg. Experimental Group2: (*n* = 7) Lavender oil at 100 mg/kg Experimental Group3: (*n* = 7) Lavender oil at 200 mg/kg	Lavender oil therapy (doses of 200 and 400 mg/kg) significantly improved functional outcomes after cerebral ischemia by diminishing brain edema, infarct size. Lavender oil at a concentration of 200 mg/kg, increased the activities of glutathione peroxidase, superoxide dismutase, and total antioxidant capacity while also reducing the content of MDA. No effect was reported on the apoptosis pathway
				**Experiment 2**:To evaluate the effect of lavender oil on brain edema, 28 animals were equally and randomly divided into four groups: Sham group: (*n* = 7) Experimental Group1: (*n* = 7) MCAo + maize oil 1 ml/kg Experimental Group2: (*n* = 7) Lavender oil at 100 mg/kg Experimental Group3: (*n* = 7) Lavender oil at 200 mg/kg Experimental Group4: (*n* = 7) Lavender oil at 400 mg/kg **Experiment 3**:To determine the effect of lavender oil on antioxidant capacity, 18 animals were equally and randomly divided into three groups: Sham Group: (*n* = 6) Experimental Group1: (*n* = 6) MCAo + maize oil, 1 ml/kg Experimental Group2: (*n* = 6) Lavender oil at 200 mg/kg **Experiment 4**:To determine the effect of lavender oil on neurotrophic activity, 18 animals were equally and randomly divided into three groups: Sham Group: (*n* = 6) Experimental Group1: (*n* = 6) MCAo + maize oil, 1 ml/kg Experimental Group2: (*n* = 6) Lavender oil at 200 mg/kg	
Chen et al. (2016)	Mice (Ischemia)	Trans-cinnamaldehyde (TCA), essential oil orally administrated 60 min before ischemia surgery	Neuroprotective effects	Mice were divided into four groups: Control group: ischemia/reperfusion induction Experimental Group1: TCA treatment at 10 mg/kg Experimental Group2: TCA treatment at 20 mg/kg Experimental Group3: TCA treatment at 30 mg/kg	Treatment with TCA essential oil strongly reduced infarct area in the cerebral ischemic area.
Rabiei et al. (2017)	Rats (Ischemia)	Lavender oil intraperitoneal injection (100 or 200 mg/kg) administrated for 20 consecutive days	Neuroprotective effects	Mice were divided into seven groups: 1) Control Group- ischemia: distilled water and underwent focal cerebral ischemia 2) Sham group: surgery without obstruction 3) Group under ischemic surgery 4) Group undergoing ischemic surgery 5) Intact-control: group without surgery; did not sit and received only distilled water 6) Intact100: No surgery 7) Intact200: No surgery	Treatment with Lavender oil at a dose of 200 mg/kg induced a significant increase of nitric oxide and a decrease in the infarct volume localized in cortical and subcortical brain regions
Huang et al. (2018)	Mice (Ischemia)	*Curcuma longa* intraperitoneal injection (200 mg/kg) administrated 30 min after brain lesion	Neuroprotective Effects	30 rats were randomly divided into six groups: 1) Control group 2) Experimental Group1: (*n* = 6); MCAO group 3) Experimental Group2: (*n* = 6); MCAO + curcumin group, 4) Experimental Group3: (*n* = 6); Y294002 + MCAO) 5) Experimental Group4: (*n* = 6); curcumin group 6) Experimental Group5: (*n* = 6); anisomycin + MCAO + curcumin group	*Curcuma longa* in significantly improved brain damage and neurological function. Curcuma exerts neuroprotective effects by attenuating autophagic activities
Amantea et al. (2009)	Mice (Ischemia)	BEO intraperitoneal injection (0.05/0.1/0.5/1 ml/k) administrated 1 h after brain lesion	Neuroprotective Effects	Male Wistar rats were used. Five experimental groups were employed: 1) Control group; 2) BEO-treated group1: concentration of 0.05 ml/kg; 3) BEO-treated group2: concentration of 0.1 ml/kg; 4) BEO-treated group3: concentration of 0.5 ml/kg; 5) BEO-treated group4: concentration of 1.0 ml/kg.	BEO reduced infarct volume in the medial striatum and motor cortex, and excitatory aspartate and glutamate, in the frontoparietal cortex

*Legend: MDA, malondialdehyde; MCAo, middle cerebral artery occlusion; VEGF, vascular endothelial growth factor; C.oil, Curcuma oil; LAF, Lavandula angustifolia flower; TCA, Trans-cinnamaldehyde, BEO; Bergamot Essential Oil*.

## Aromatherapy in Stroke Patients: First Evidence

Considering clinical stroke patients, only three studies have directly evaluated the pure effect of aromatherapy. However, in all clinical studies evidence on a better outcome after treatment has been reported, mainly considering pain, motor, and autonomic responses. In particular, Shin and Lee (2007) used aromatherapy in the participants with hemiplegic shoulder pain (HSP) after stroke. Thirty subjects were equally and randomly assigned to acupressure-only control group or aromatherapy acupressure experimental group. All treatments consisted of 20-min sessions twice-daily for 2 weeks, giving a total of 28 treatments. In the experimental group, patients underwent aromatherapy acupressure with essential oils of lavender, rosemary, and peppermint in a 2:1:1 ratio diluted to 3% in jojoba oil. The control group underwent dry acupressure at the same acupuncture points. Authors claimed that aromatherapy acupressure significantly reduces HSP in stroke patients with respect to acupressure alone. However, both interventions are effective at improving motor response. However, the authors suggest further randomized and follow-up studies to clarify whether the beneficial effect of aromatherapy acupressure persists for the long-term on psychological and physiological characteristics related to HSP. Salehi et al. (2019) investigated the effect of aromatherapy with lavender 10% essential oil on psychological, motor, and cognitive functions in acute thrombotic cerebral ischemic patients. Seventy patients were equally and randomly assigned to experimental or control groups. In the control group, patients received routine medical treatment (such as enoxaparin, aspirin, atorvastatin, and antihypertension medications) together with a placebo instead of lavender essential oil. While the experimental group received routine medications in addition to aromatherapy. All treatments consisted of daily sessions over 1 week. The intervention group showed improvement in several symptoms, such as motor function, speech, and delirium with respect to the control group. This positive effect was associated with a reduction of oxidative stress and an increase in the level of antioxidants. Finally, Lee et al. (2017) evaluated the clinical effects of aromatherapy massage. Treatments were made five times in 1 week with each session lasting for 30 min. The essential oils included a combination of juniper, lavender, orange, patchouli, and rosemary. The main outcomes were stress level, body temperature, mood, and quality of sleep. Fourteen stroke patients were equally and randomly divided into two groups of patients: Control Group (*n* = 7), who underwent general physical therapies, and Experimental Group (*n* = 7), who performed general physical therapies together with food bath and aromatherapy massage. These authors described significant improvement in psychological stress, mood status, and quality of sleep together with lower body temperature.

## Summary

Despite the paucity of studies on this topic (mainly in the clinical realm) this perspective article is not meant to be an inclusive review of research on aromatherapy, but to provide the reader with our view of this new challenging field, highlighting some research areas that could be of interest and concluding with a summary of where we think the field might go in the future.

Evaluating data from preclinical trials, it emerges that aromatherapy produces a consistent effect as a therapeutic agent for the prevention of ischemic brain damage promoting neuroprotective activities. In accordance, preliminary human studies confirmed that the effects of essential oils go beyond the neurobiological domain influencing also the somatic and behavioral status of stroke patients. Obviously, the animal model offers the opportunity to better disentangle the impact of essential oils systematically and rigorously with respect to human studies, and this is mandatory to determine the pharmacological basis of aromatherapy (Scuteri et al., 2017b).

As showed in Table 1, all preclinical studies reported significant and robust impact of essential oils (including Curcuma, Bergamot Lavender, TCA oils) in reducing infarct size, brain edema, neural death/damage, exerting a neuroprotective effect by increasing the antioxidant defense, activities of superoxide dismutase, glutathione peroxidase, and reducing apoptotic cells, nitric oxide synthase, autophagic activities, and the content of MDA (Büyükokuroğlu et al., 2003; Dohare et al., 2008; Amantea et al., 2009; Wang et al., 2012; Vakili et al., 2014; Chen et al., 2016; Rabiei et al., 2017; Huang et al., 2018). Among the essential oils, it emerged that Curcuma longa has been associated with a reduction of NOS isoforms, inhibition of caspase-3 concurrently with activation of Bcl-2 pathways (instead of lavender implicated mainly in oxidative balancement), and modulation of autophagy. The latter evolutionarily conserved process is also involved in the pharmacological action of BEO. Indeed, BEO can counteract the efflux of excitatory amino acids and the production of ROS reverting the functioning of glutamate transporters occurring under ischemic conditions. However, the effects of essential oils extend beyond the anti-oxidant properties described in the animal model of stroke (Ramsey et al., 2020). In particular, BEO has also provided strong evidence of anti-nociceptive, anti-neuropathic, and anxiolytic-like flumazenil-insensitive properties in preclinical models (Scuteri et al., 2018a, 2019; Rombolà et al., 2019), forming the rational basis for the clinical study on agitation and pain in dementia in the pharmaceutical form of a smell-devoid nanotechnological cream, i.e., NanoBEO (Scuteri et al., 2021a). This will allow for the first time a double-blind clinical trial (NCT04321889; Scuteri et al., 2021b) assessing the efficacy and safety of a demonstrated analgesic essential oil in patients suffering from Alzheimer’s disease, whose pain and behavioral and psychological symptoms are often inappropriately treated also in the community setting (Gok Metin et al., 2017; Scuteri et al., 2017a, 2018b, 2020, 2021a,b,c). This clinical trial can pave the way for the following studies assessing the efficacy and safety of NanoBEO with observational pain assessment tools in patients with stroke and in critically ill patients affected by chronic pain, a population intensely growing due to the Coronavirus disease COVID-19 pandemic (Scuteri et al., 2020).

The management of pain is mandatory for the care of stroke patients. Pain after stroke is a symptom commonly reported but often incompletely managed. It can be easily overlooked due to its variable characteristics, concurrent comorbid medical issues, or impairments in cognition or communication. While pain can create its own disabilities secondary to a decrease in function, its effect on the recovery of post-stroke patients can substantially impact a patient’s future quality of life. Evidence provided by Shin and Lee (2007) confirmed that in the stroke domain also one of the most important beneficial effects of aromatherapy is the effective pain reduction, as already demonstrated in other neurological domains, such as neuropathic pain, chronic neck pain, episiotomy pain, post-cesarean section pain, postoperative pain, renal colic, and Guillain-Barré Syndrome (Lakhan et al., 2016; Gok Metin et al., 2017). Aromatherapy could also be less expensive and has fewer side effects than traditional pain management drugs. Evidence coming from the human studies would suggest the definition of new randomized controlled clinical trials to amplify this effect useful to provide stability and strain relief. In this regard, the works made by Lee et al. (2017) and Shin and Lee (2007) demonstrated that the effects of aromatherapy extend beyond the pain domain. Indeed, a better improvement after treatment has been found for behavioral symptoms (stress level, sleep quality, delirium) as well as for clinical symptoms (motor and language). Despite this, it must be considered only as preliminary evidence, however, part of these findings agrees with previous findings reported for other neurological disorders, such as Alzheimer’s disease, where aromatherapy consistently has been applied to reduce agitation, aggression, and psychotic symptoms (Jimbo et al., 2009; Iranshahi and Javadi, 2019; Banerjee et al., 2021).

In conclusion, despite aromatherapy application in stroke patients being at its very infancy, the consistent results obtained from preclinical trials and the preliminary work on the human models would seem to encourage its translation from bench to bedside. Despite the small number of studies, it emerges that essential oils possess anti-inflammatory, and antioxidant properties as well as purported psychological effects that may alleviate somatic and emotional diseases. However, in human studies, the appropriate dosage or procedure needs to be standardized. For this reason, it is crucial that future research should focus on the pharmacological profile of specific essential oil agents to establish phytochemical standardization, mechanism of action, and any clinical contraindications. Moreover, the combination of genetic and biological measurements on stroke patients after aromatherapy treatment will allow us to bridge the gap between preclinical evidence and clinical outcome. Overall, we believe that aromatherapy has the potential to become a complementary method to treat behavioral and emotional disorders in stroke, as already done for other clinical disorders (Hines et al., 2012; Candy et al., 2020), but additional clinical studies are mandatory.

## Author Contributions

MC and AC: conceptualization, writing—original draft preparation, writing—review and editing, visualization. PT, GB, and DS: supervision. All authors contributed to the article and approved the submitted version.

## Conflict of Interest

The authors declare that the research was conducted in the absence of any commercial or financial relationships that could be construed as a potential conflict of interest.

## Publisher’s Note

All claims expressed in this article are solely those of the authors and do not necessarily represent those of their affiliated organizations, or those of the publisher, the editors and the reviewers. Any product that may be evaluated in this article, or claim that may be made by its manufacturer, is not guaranteed or endorsed by the publisher.
